# Supplementary material for: Role and mechanism of NCAPD3 in promoting malignant behaviors in gastric cancer
Source: Front Pharmacol. 2024 Apr 22;15:1341039. doi: 10.3389/fphar.2024.1341039 (PMC11070777; doi:10.3389/fphar.2024.1341039)
Supplement: Supplementary file 11 [file DataSheet2.ZIP › GSEA/GO_CC/my_analysis.Gsea.1599461408869/GO_ACTIN_BASED_CELL_PROJECTION.html]

Details for gene set GO\_ACTIN\_BASED\_CELL\_PROJECTION[GSEA]

|  || Dataset | filtered\_dataset.sample\_info.cls#WT\_versus\_NCAPD3\_MUT |
| Phenotype | sample\_info.cls#WT\_versus\_NCAPD3\_MUT |
| Upregulated in class | NCAPD3\_MUT |
| GeneSet | GO\_ACTIN\_BASED\_CELL\_PROJECTION |
| Enrichment Score (ES) | -0.42119 |
| Normalized Enrichment Score (NES) | -1.8702986 |
| Nominal p-value | 0.012254902 |
| FDR q-value | 0.06527888 |
| FWER p-Value | 0.256 |
Table: GSEA Results Summary

  

Fig 1: Enrichment plot: GO\_ACTIN\_BASED\_CELL\_PROJECTION      
 Profile of the Running ES Score & Positions of GeneSet Members on the Rank Ordered List

  

| SYMBOL | TITLE | RANK IN GENE LIST | RANK METRIC SCORE | RUNNING ES | CORE ENRICHMENT || 1 | 7976 | FZD3 | 88 | 0.815 | 0.0080 | No |
| 2 | 538 | ATP7A | 305 | 0.588 | -0.0958 | No |
| 3 | 960 | CD44 | 339 | 0.573 | -0.0694 | No |
| 4 | 4771 | NF2 | 428 | 0.516 | -0.0876 | No |
| 5 | 3093 | UBE2K | 795 | 0.326 | -0.3220 | No |
| 6 | 1605 | DAG1 | 823 | 0.304 | -0.3148 | No |
| 7 | 7429 | VIL1 | 827 | 0.299 | -0.2908 | No |
| 8 | 26031 | OSBPL3 | 870 | -0.281 | -0.2964 | No |
| 9 | 10123 | ARL4C | 957 | -0.377 | -0.3252 | No |
| 10 | 55591 | VEZT | 973 | -0.389 | -0.3020 | No |
| 11 | 6286 | S100P | 1011 | -0.412 | -0.2926 | No |
| 12 | 5154 | PDGFA | 1191 | -0.566 | -0.3717 | Yes |
| 13 | 4651 | MYO10 | 1213 | -0.588 | -0.3354 | Yes |
| 14 | 2043 | EPHA4 | 1228 | -0.602 | -0.2928 | Yes |
| 15 | 55 | ACPP | 1265 | -0.656 | -0.2613 | Yes |
| 16 | 57348 | TTYH1 | 1306 | -0.710 | -0.2280 | Yes |
| 17 | 5794 | PTPRH | 1368 | -0.854 | -0.1972 | Yes |
| 18 | 5156 | PDGFRA | 1383 | -0.932 | -0.1258 | Yes |
| 19 | 760 | CA2 | 1410 | -1.654 | 0.0000 | Yes |
Table: GSEA details [plain text format]

  

Fig 2: GO\_ACTIN\_BASED\_CELL\_PROJECTION      
 Blue-Pink O' Gram in the Space of the Analyzed GeneSet

  

Fig 3: GO\_ACTIN\_BASED\_CELL\_PROJECTION: Random ES distribution      
 Gene set null distribution of ES for **GO\_ACTIN\_BASED\_CELL\_PROJECTION**
